# Renewable Syngas Generation via Low-Temperature Electrolysis: Opportunities and Challenges

**DOI:** 10.1021/acsenergylett.3c02446

**Published:** 2023-12-29

**Authors:** Andrés Raya-Imbernón, Angelika A. Samu, Stefan Barwe, Giuseppe Cusati, Tamás Fődi, Balázs M. Hepp, Csaba Janáky

**Affiliations:** † Air Liquide Forschung & Entwicklung GmbH, Innovation Campus Frankfurt, Gwinnerstraße 27−33, 60388 Frankfurt am Main, Germany; ‡ eChemicles Zrt, Alsó Kikötő sor 11, Szeged H-6726, Hungary; § Department of Physical Chemistry and Materials Science, University of Szeged, Rerrich Square 1, Szeged H-6720, Hungary

## Abstract

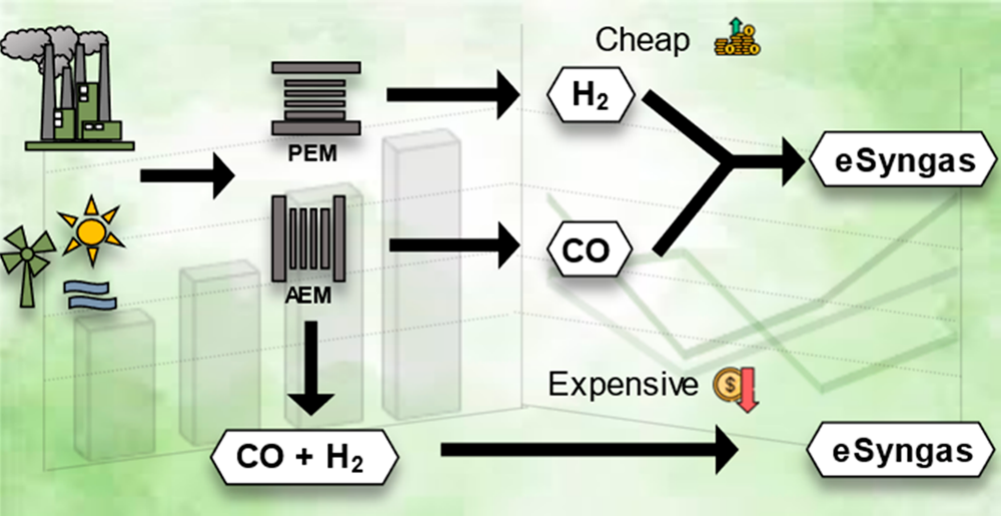

The production of syngas (i.e., a mixture of CO and H_2_) via the electrochemical reduction of CO_2_ and water can contribute to the green transition of various industrial sectors. Here we provide a joint academic–industrial perspective on the key technical and economical differences of the concurrent (i.e., CO and H_2_ are generated in the same electrolyzer cell) and separated (i.e., CO and H_2_ are electrogenerated in different electrolyzers) production of syngas. Using a combination of literature analysis, experimental data, and techno-economic analysis, we demonstrate that the production of synthesis gas is notably less expensive if we operate a CO_2_ electrolyzer in a CO-selective mode and combine it with a separate PEM electrolyzer for H_2_ generation. We also conclude that by the further decrease of the cost of renewable electricity and the increase of CO_2_ emission taxes, such prepared renewable syngas will become cost competitive.

## The Importance of Syngas

For decades, hydrogen (H_2_) and carbon monoxide (CO) have been used in a variety of ways as building blocks for chemical and fuel production. Depending on the final product and the type of process, H_2_ and CO can be utilized separately or together. When combined, their mixture is commonly known as syngas. Nowadays, syngas is mainly produced via either reforming or partial oxidation of fossil resources, such as natural gas, naphtha, and heavy residual fuel oils. Other methods include gasification of coal, and most recently of municipal solid waste and biomass.^1^ The methods using fossil resources are energy intensive with huge environmental footprint, including significant carbon dioxide (CO_2_) emissions.^2^ The biomass-based processes, on the other hand, are limited in scale. The type of feedstock and the associated production process determine the syngas ratio (i.e., the ratio of H_2_ and CO). A specific ratio is needed depending on the application, such as the production of pharmaceuticals, plastics, chemicals, or synthetic fuels (Tables 1 and S1).^3−6^

**Table 1:** Illustration of the H_2_:CO Ratio Required for the Main Syngas Applications

H_2_:CO Ratio	Application
<1	Polyurethanes, polycarbonates, acetic acid
1	Oxo alcohols, dimethyl ether
2	Methanol, Fischer–Tropsch liquid fuels

The global syngas market has been estimated to have a size of 218 MM Nm^3^/h in 2022 including hydrogen and ammonia, using syngas as an intermediary.^7^ The market is projected to increase notably in the coming decades (with an approximate CAGR of 6% by 2028).^7^ This increase is rooted in the projected growth of industries consuming syngas today, as well as the fact that novel pathways of utilization are expanding.^8,9^ To fight global warming and to comply with the Paris Agreement (COP-21) and consequent regulatory changes (e.g., RED III in Europe^10^), massive greenhouse gas emission reductions in all sectors are required by 2050.^11^According to The International Energy Agency (2021), to meet current net-zero targets, the rapid deployment of appropriate carbon capture and utilization (CCU) technologies is required to stay on track with carbon emissions by 2050. Consequently, the production of low-carbon syngas on CO_2_ basis, together with electrification and hydrogen mobility, is expected to significantly contribute to the decarbonization of both industrial and transportation sectors.^12−16^

**Figure 1:**
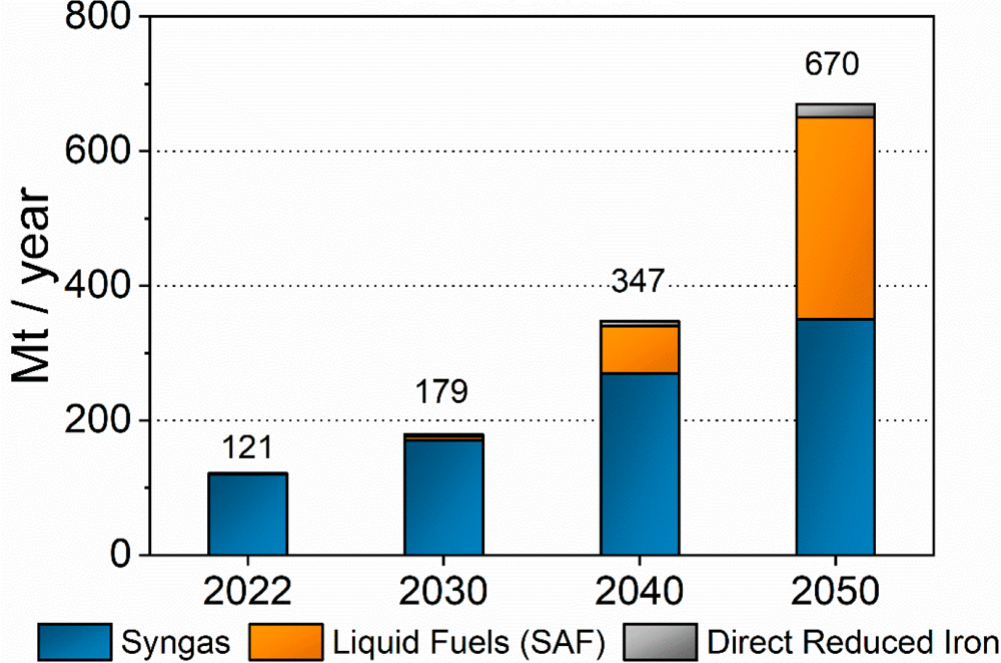
Long-term prediction of the syngas market size (own estimation based on the growth of the key market segments, see section S5 in the Supporting Information).

In Figure 1 we illustrate the growth potential of syngas for the different chemical and fuel markets (excluding all hydrogen and ammonia production and energy applications). We specifically show that the growth is expected to come mainly from the synthetic sustainable fuels market which currently is in its infancy. Notably, it is very difficult to estimate the current syngas (CO + H_2_) market size because most statistics and analysis include all sorts of synthetic gases in this category (e.g., N_2_ + H_2_ mixtures for ammonia synthesis). Our approach was to analyze the markets and market trends of the most important syngas-derived products (see the Supporting Information).

## Electrochemical Syngas Generation

Traditionally, CO has been produced in large, centralized plants that profit from the economy of scale. Its toxicity and flammability, however, make the transportation of pressurized CO gas cylinders or tube trailers very hazardous and therefore expensive. Electrolysis, when using electricity from renewable sources, is one of the most prominent and environmentally friendly solutions to produce such chemicals.^17^ Water electrolysis to produce renewable hydrogen has reached commercial maturity, and large projects have been announced to get into operation in the coming years.^18^ CO_2_ electrolysis is another promising method, allowing the electrochemical conversion of CO_2_ to chemicals.^19^ From an energetic point of view, the production of CO is the most favorable large market chemical that can be obtained via CO_2_ electrolysis.^20,21^ Electrochemical processes can be operated at low temperatures and pressures, as opposed to other chemical or catalytic processes. Furthermore, it allows decentralized operation, because the scale has little effect on the system cost and efficiency. Another key differentiator compared to traditional methods is the possibility of dynamic operation, as we have recently demonstrated.^22^ Overall, the electrochemical route would make CO production not only more sustainable but also less centralized/more distributed and therefore suitable for a larger number of applications.

According to different techno-economic assessments (TEAs), electrochemical production of CO can already be profitable, if the technology is available at scale.^20,21,23^ Furthermore, for applications where a steady supply of syngas is not needed, the electrochemical reduction of CO_2_ provides a unique opportunity to convert intermittent but abundant renewable energy source into chemical fuels.^24^ What is equally important, different life cycle assessment (LCA) studies confirmed that such electrochemical routes can have a significant reduction in CO_2_ footprint, compared to the traditional methods.^25,26^ Meeting these three conditions together (i.e., economic viability, CO_2_ footprint decrease, and large market size) predicts a great promise for electrochemical CO_2_-to-CO conversion. At the same time, several challenges, such as low energy efficiency, short demonstrated system lifetime, and consequently high capital and operating costs, must be overcome prior to commercialization. Such efforts are underway at several companies.^19,27^

Due to the presence of water in the electrolyzer system (vapor or liquid, depending on the reaction conditions and the electrolyzer type), part of the electrical current applied for the CO_2_ electrolysis may generate H_2_ instead of CO, lowering the Faradaic efficiency (FE) of CO formation. This opens the opportunity for the one-step generation of syngas, which is often claimed as a benefit of such technologies. A recent article analyzed the economics of electrochemical syngas production, comparing different processes and cell configurations, with particular emphasis on the integration of direct air capture.^28^ Our Perspective focuses on a different angle of the story, namely, the separate vs concurrent production of the two components of syngas (i.e., H_2_ and CO).

## Options for Green Syngas Generation

Several types of CO_2_ electrolyzer setups can be used for renewable syngas production, each of them having its own advantages and limitations.^29^ High-temperature solid-oxide electrolyzers have been recently benchmarked against conventional CO-generating processes.^30^ This Perspective focuses on low-temperature electrolysis because, as opposed to the high-temperature processes, such systems can be operated dynamically and under similar conditions as proton exchange membrane (PEM), alkaline, and anion exchange membrane (AEM) water electrolyzers. More specifically, we investigated the AEM containing gas diffusion electrode (GDE) system for both CO- and syngas production from CO_2_. Such cells employ a GDE to enhance the mass transport of CO_2_ to the catalyst, resulting in higher current density and single-pass conversion.^31,32^ AEM CO_2_ electrolyzers show better energy efficiencies and often use less expensive materials, compared to their PEM-based and bipolar membrane-containing counterparts.^33^ Carbonate formation, however, is a challenge for this technology, as carbonate/bicarbonate ions are formed at the cathode when CO_2_ comes into contact with OH^–^ ions.^34^ Due to the unintended cation crossovers from the anolyte,^32,35^ carbonates can precipitate, leading to accumulation and poisoning/flooding of the cathode GDE. Carbonate ions also migrate to the anode, leading to the coevolution of CO_2_ and O_2_ and thus to more purification efforts.^34,36^

**Figure 2:**
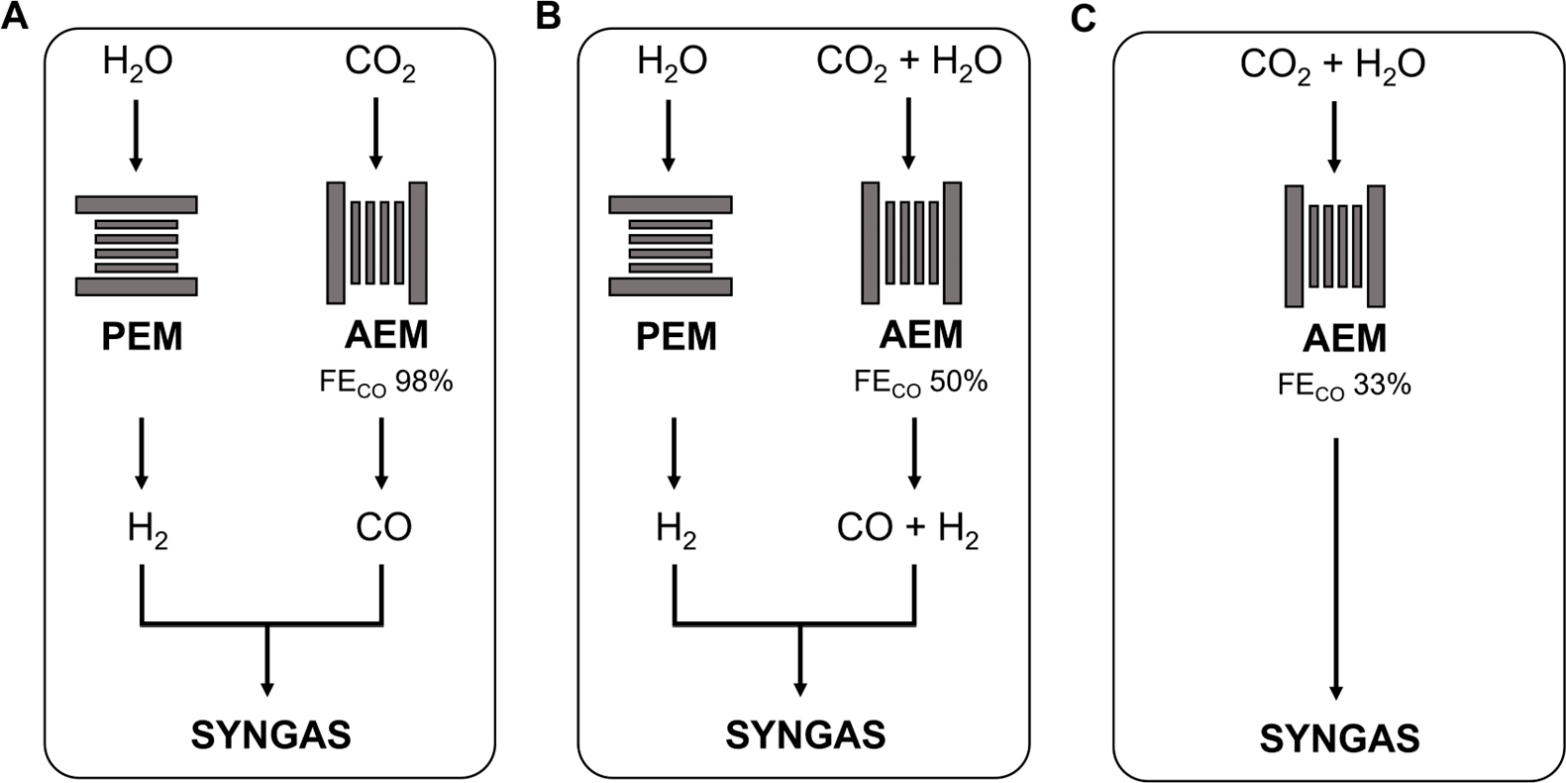
Illustration of the three different syngas production scenarios analyzed in this Perspective.

In this study, we studied three options to produce syngas with an H_2_:CO ratio of 2:1 (Figure 2):

A: AEM CO_2_ electrolyzer with a FE of 98% toward CO. The final syngas is obtained by mixing the CO with H_2_ produced from a PEM water electrolyzer. The syngas composition can then be controlled by adjusting the relative proportions of CO and H_2_.
B: AEM CO_2_ electrolyzer with a FE of 50% toward CO. The resulting H_2_:CO ratio of 1:1 is then modified by the addition of H_2_ coming from a PEM water electrolyzer.
C: AEM CO_2_ electrolyzer with a FE of 33% toward CO. The desired syngas composition with an H_2_:CO ratio of 2:1 is obtained using only the AEM CO_2_ electrolyzer. The FE and consequently the syngas composition can be adjusted by varying the operational parameters of the electrolyzer (e.g., cell voltage, CO_2_ and H_2_O feed rate).

### Technical Considerations

CO_2_ electrolysis literature has grown massively during the past two decades. Many of these papers claim “the production of syngas with tunable composition” as their key selling point. This has been seen for Ag, Au, Zn, different bimetallic, Pd hydride, and metal–nitrogen doped carbon catalysts.^37−42^ These catalysts are known to form CO as the predominant CO_2_ reduction (CO2R) product, with the concurrent formation of H_2_. In Table S1, we summarize selected examples from the literature, where the H_2_:CO ratio varied between the broad range of 1:4 to 25:1 (a range of 2 orders of magnitude(!)). This variation was mostly attributed to the catalyst surface composition and the applied electrode potential. While these exploratory studies are interesting, they were performed in H-cells, in the presence of one or more aqueous electrolytes (i.e., one electrolyte in membrane-less cells and two electrolytes (anolyte and catholyte) in membrane-separated cells), at low current density (up to 20 mA/cm^2^), for short time periods (typically up to a few hours).

From a practical perspective, the most promising studies for CO and syngas generation have been reported on GDE-containing cells and stacks.^19,43^ The results are massively different from those obtained in H-cells. This is mainly because the selectivity (i.e., HER vs CO2R ratio) is dictated by multiple parameters beyond the catalyst itself: components of the membrane electrode assembly (MEA), local chemical environment (pH, water content), and operational parameters (gas flow rate, etc.). There are only a very few long-term studies for CO formation,^44−47^ and no long-term data is available for syngas generation. We think this is partly because the most important fading mechanism in such systems is flooding when too much electrolyte accumulates in the cathode GDE. This in turn results in increased HER, which ultimately leads to cell fading. Some studies indicate the intricate connection among precipitate formation, flooding, and increased HER,^48,49^ but it is not within the scope of this Perspective. There is, however, reason to believe that process conditions in which large amounts of H_2_ are generated favor the eventual flooding of the cathode.

**Figure 3:**
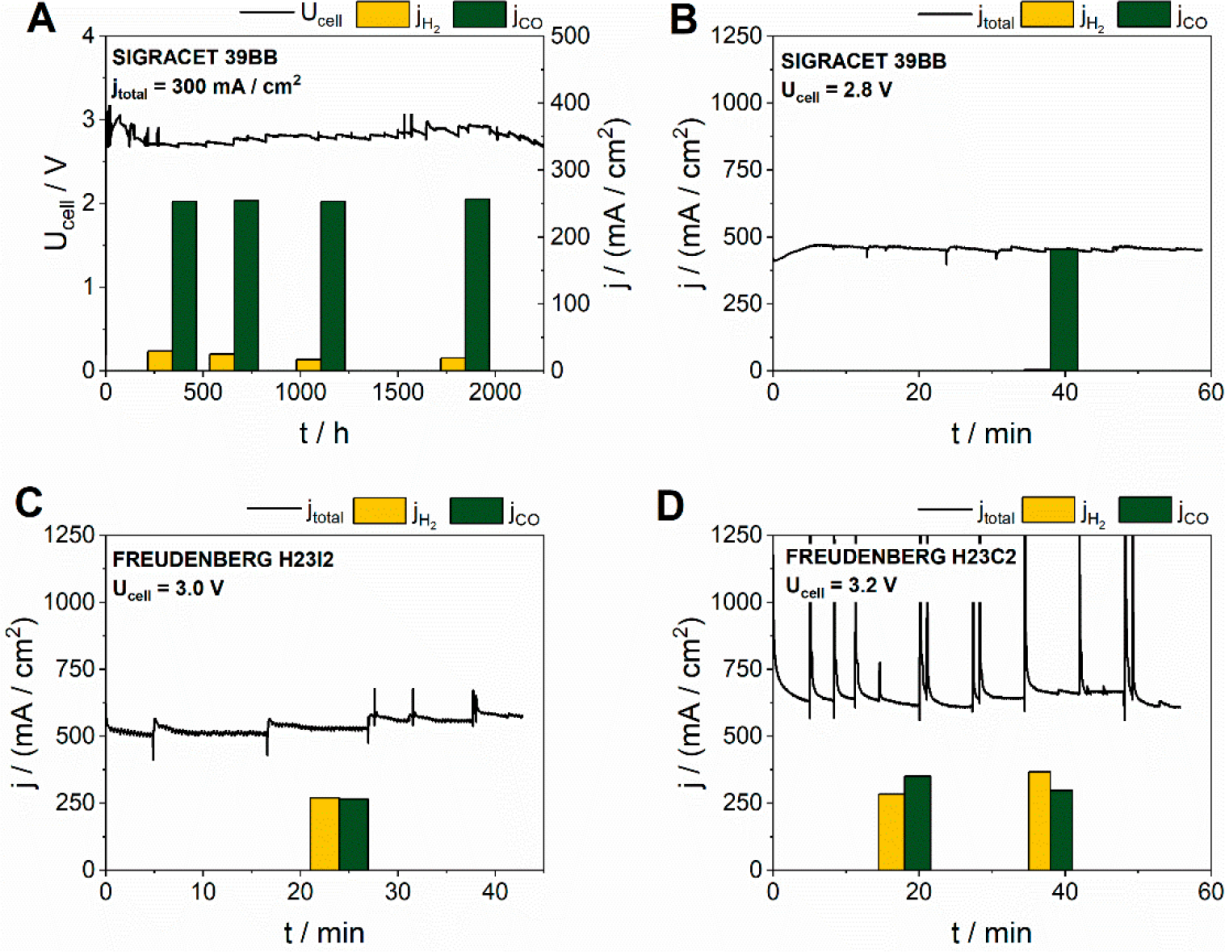
Illustrative electrochemical data for the formation of CO and syngas formation. (A, B) An optimized zero-gap electrolyzer cell; (C, D) nonoptimal carbon GDLs. Results were obtained with a zero-gap electrolyzer cell with Ag cathode, Ir anode, and PiperION anion exchange membrane, using 0.05 M (A) or 0.1 M (B–D) CsHCO_3_ anolyte.

In Figure 3A we show our own data as an example of the long-term stable operation of a CO-selective CO_2_ electrolyzer (with over 90% FE for CO formation for over 2000 h, with an approximate degradation rate of ∼50 μV/h) and examples for the other target gas compositions, obtained using GDEs with different structures. Clearly, the use of Sigracet 39BB carbon GDL, together with proper electrolyte management (to keep steady-state local pH and cation concentration), results in a CO-selective operation (Figure 3B), while the use of Freudenberg H23I2 and Freudenberg H23C2 carbon GDLs results in an increased HER activity (see more measurements on different GDLs^37^). Overall, depending on the cell components and the applied cell voltage, we can get a similar product composition as in the three targeted syngas production pathways previously defined (Figure 2). At the same time, the more frequent and larger spikes on the current curves with the increasing H_2_ content indicate pronounced water accumulation and release in the cell, which is typically the first step on the way to flooding. In addition to our own data, we present a brief comparison of selected studies from the literature where high current densities were obtained (Table S1).

Based on the above literature analysis, our own data, the historic learning curve of PEM and AEM water electrolyzers, and future predictions, we defined today’s and future (2030) operational parameters for the three studied scenarios (Table 2). These numbers also reflect the expected effects of ongoing engineering efforts, integrating state-of-the art cell components, implementing methods established by allied fields (e.g., MEA production in the fuel cell industry). Please note that with regard to the single-pass conversion, an exclusive carbonate ion transport from the cathode to the anode has been assumed for all the cases, including scenarios “B” and “C” where the HER becomes prevalent (i.e., the charge carrier species are still the carbonate ions; see more discussion in Table S2).

**Table 2:** Electrochemical Performance Parameters for Different Cases

	CO_2_ Electrolyzer						
	Case A		Case B		Case C		PEM Electrolyzer
	Today	2030	Today	2030	Today	2030	Cases A and B
FE_CO_ (%)	98%	98%	50%	50%	33%	33%	0%
Current density (mA/cm^2^)	500	600	800	900	1000	1100	2000
Voltage (V)	2.6	2.4	2.9	2.8	3.1	3.0	1.9
Single-pass CO_2_ conversion (%)	40%	40%	25%	28%	15%	18%	0%
Degradation rate (μV/hours)	30	10	50	25	80	40	2.6

## Technoeconomic Comparison

To evaluate the viability of the three different green syngas production routes, and to provide guidance to the R&D community, the total cost of ownership (TCO) of the syngas (expressed in €/kg_SG_) has been calculated. The main methods and governing equations used in this paper are in accordance with the ones described in previous CO_2_ electrolysis TEA studies (see details in the Supporting Information).^20,23^ Nowadays, a typical industrial capacity for CO production only is 10,000 Nm^3^/h and is usually supplied through reforming of natural gas in which large quantities of hydrogen are also coproduced.^50^ The same CO capacity has therefore been set for the CO_2_ electrolysis as benchmark. As the desired H_2_:CO ratio of syngas in this study is 2:1, the final syngas quantity to be produced will be 30,000 Nm^3^/h (342 t/day).

As low-temperature CO_2_ electrolysis has not reached commercial availability yet, the CAPEX of the CO_2_ electrolyzer stack has been extrapolated from the cost of the more mature water electrolysis (see Table S3 for our literature review).^51^ In parallel, a bottom-up approach has also been performed to estimate stack component prices (Table S4). The final cost of the CO_2_ stack used for this study was 2587 €/m^2^. This value should be seen as a target cost in 2030 for a 10 MW stack (at a reference voltage and current of 2.6 V and 500 mA/cm^2^) rather than as a current stack cost. The overall cost of the system is then obtained by adding the balance of plant costs (the stack contributes ca. 30% to the total system cost, with the other 70% being the balance of the plant^52^). An installation factor of 1.6 is used for the electrolyzer system.^53^ The resulting total installed cost of a CO_2_ electrolyzer is therefore 13797 €/m^2^. The complementary PEM water electrolysis system used to produce any hydrogen needed depending on the route considered has a total installed cost (TIC) of 1300 €/kW with an expected cost decrease of 20% by 2030.^54^ This system produces hydrogen at 56 kWh/kgH_2_ with an estimated degradation rate of 2.6 μV/hour.^55,56^ Due to degradation, the stacks need to be replaced over the lifetime of the system.^56,57^ For each configuration and for both water and CO_2_ electrolyzers, the optimum number of stack replacements was calculated to minimize the cost (see the Supporting Information). What is often neglected in academic TEA studies is the fact that the rectifiers and power electronics are designed to function within specific voltage limits.^58^ A significant voltage increase beyond this range can induce various technical challenges, including reduced system efficiency, increased wear and tear on equipment, and potential safety risks.^59^ To avoid these issues, the electrolyzer stack in this study is assumed to be replaced before a 50% voltage increase due to degradation is reached.

To limit CO_2_ consumption and therefore variable costs (and also to achieve the maximum CO_2_ emission avoidance), CO_2_ capture and recirculation is needed both at the cathode stream (because of the incomplete CO_2_ single-pass conversion) and at the anode stream (because of carbonate crossover and subsequent CO_2_ liberation).^34,60^ In CO_2_ capture from gaseous streams, various methods can be employed, including chemical absorption, cryogenic, and membrane separation.^61^ Pressure swing adsorption (PSA), in which CO_2_ is selectively removed from the gaseous mixture using solid adsorbents, has been used for this study at both the anode and cathode sides. This is due to its low energy consumption, its broad adaptability to different capture needs, and its ability to achieve high purity levels.^62^ For this study the technology taken as a reference is the PSA used in biogas upgrade.^63^ A scaling factor of 0.7 was used to adjust the reference costs to our system.

The two main variable costs considered are renewable electricity and the CO_2_ feedstock. As the Fischer–Tropsch process requires a continuous and stable feed supply to produce liquid fuels, the renewable electricity used to produce H_2_ and CO must be purchased at a very high availability. Different studies project levelized costs of energy (LCOE) for utility-scale PV and wind in 2030 between 20 and 35 €/MWh; however, such power comes with high intermittency and low capacity factors (20–40%).^64−66^ To maintain a high availability, a combination of PV, wind, and energy storage is necessary.^67^ This configuration, however, will increase the overall electricity cost due to the need for excess capacity and storage infrastructure.^68^

For renewable syngas to be considered a real carbon sink, the CO_2_ must come from direct air capture (DAC) or biogenic sources. DAC’s current high cost makes it less economically attractive for large-scale fuel production.^69^ On the other hand, biogenic CO_2_ comes at lower costs with various sources such as fermentation, anaerobic digestion, and biomass postcombustion processes, and we used such data (purification needs of different sources may vary (e.g., SO_*x*_/NO_*x*_); that is why a relatively high average CO_2_ cost is considered).^70^ The use of CO_2_ from an industrial point-source, although largely available at a low price, would only lead to delayed emissions in the case of fuel production, and it is therefore not considered in this study.

Although use cases may exist where CO and O_2_ could be utilized by one or different end users at the same location (i.e., in oxyfuel combustion), the oxygen produced at the anode side is not valorized in the model because its cost would depend on its final purity, which in turn would need a more detailed assessment. In addition, the oxygen sale is not expected to affect the final TCO significantly. No CO_2_ tax savings or subsidies have been taken into consideration. Any other operating costs such as water consumption, adsorbent costs, and other various utilities are not considered in this study, since they would account for a very minor cost share of the TCO. The list of assumptions is summarized in Table 3.

**Table 3:** Main Process, Market, and Production Assumptions

Assumption	Value
Syngas capacity (Nm^3^/h)	30,000
H_2_:CO ratio (v/v)	2
Electricity price (€/MWh)	40
Electricity availability (%)	98
CO_2_ price (€/ton)	60^70^
Electrical consumption PSA (kWh/Nm^3^)	0.25^62^

## TEA Results

**Figure 4:**
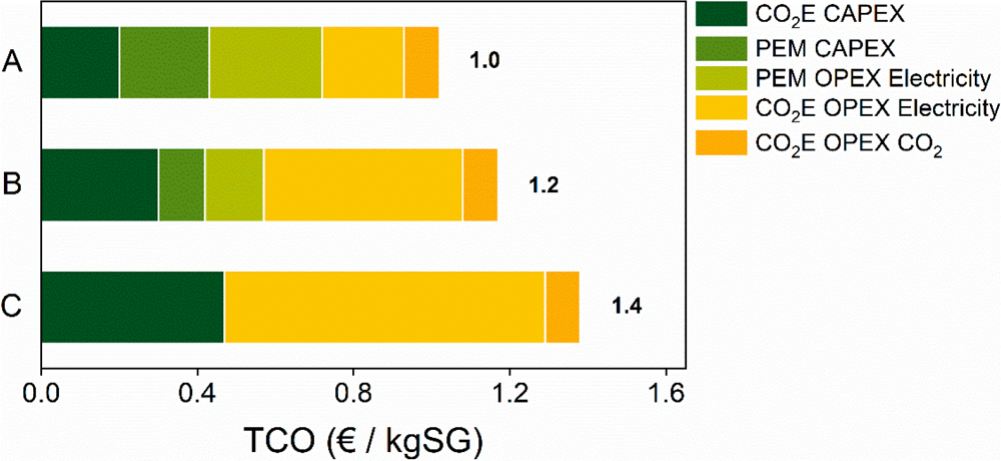
Syngas (H_2_:CO = 2:1) TCO [€/kg] for CO_2_-CO (A), CO_2_-SG-50 (B), and CO_2_-SG (C) cases in 2030.

Clearly, the most economical way of producing syngas by means of CO_2_ electrolysis is to couple a very CO-selective CO_2_ electrolyzer with a water electrolyzer delivering the required hydrogen (Figure 4). This configuration results in a final syngas price of 1 €/kgSG, which is 30% lower than the configuration in which the syngas is fully produced from a single CO_2_ electrolyzer operated at low FE_CO_. The TCO of syngas is mainly driven by the variable costs (i.e., electricity), and their contribution increases when decreasing the FE_CO_ of the CO_2_ electrolyzer. In fact, while case A produces syngas at 12.6 kWh/kgSG, the direct syngas production requires 20.5 kWh/kgSG (throughout the lifetime of the plant; Figure S3a). This difference is mainly due to hydrogen production, highlighting that the hydrogen produced during the electrolysis of CO_2_ cannot be considered to be free. The notable difference in the overpotential between AEM CO_2_ and PEM water electrolyzers (2.4 V in 2030 instead of 1.9 V) causes the hydrogen coming from a CO_2_ electrolyzer to have a higher energy cost (also reflected in Figure S3b). A PEM electrolyzer produces hydrogen over the lifetime of the system at 60 kWh/kgH_2_ while a direct syngas CO_2_ electrolyzer does it at 110 kWh/kgH_2._

The degradation rate of the electrolyzer also plays an important role in the energy efficiency of the system (Figure S3b,c). At the beginning of life, there is not a huge difference among the three different syngas production configurations. The degradation rate of the CO_2_ electrolysis, at this stage of technological immaturity, however, is much higher than that of a PEM water electrolyzer over the entire lifetime of the system. As a result, the energy consumption of hydrogen of system C (CO_2_ electrolyzer only) ends up being 90% higher than that of case A and 36% higher than case B. Notably, this is a parameter where the largest improvement is expected beyond 2030 and also needed for commercialization. The impact of the higher degradation rate of the CO_2_ electrolysis on the final syngas TCO is also reflected in CAPEX, as the stack must be replaced more often. In addition, the increased capacity requirement of PSAs brings a non-negligible additional cost to recirculate CO_2_ which gets more prominent as hydrogen is produced with the CO_2_ electrolyzer.

## Sensitivity Analysis and Pessimistic–Realistic–Optimistic Scenarios

**Figure 5:**
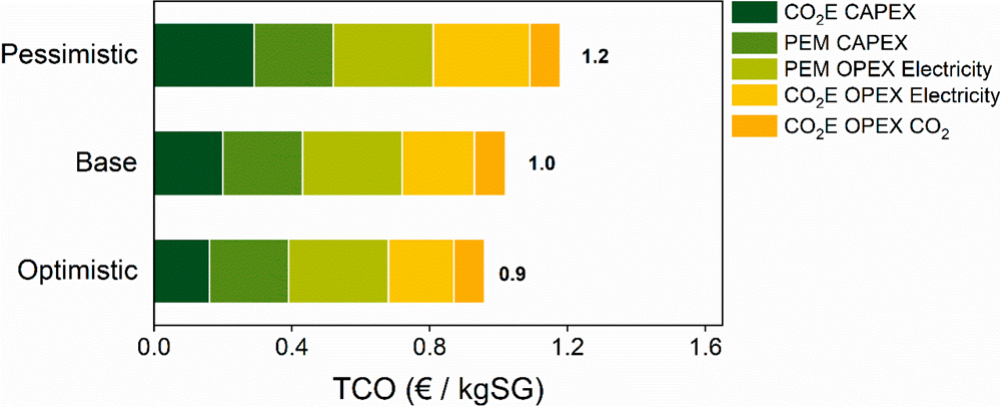
Syngas (H_2_:CO = 2:1) TCO [€/kg] for CO_2_-CO in the pessimistic, base, and optimistic cases.

A sensitivity analysis on the *operating conditions* has been carried out for this best approach (Table S7). Figure 5 shows that the price of syngas using case A can decrease to values below 1 €/kgSG. Furthermore, even assuming a pessimistic performance, Case A would remain more advantageous from an economical point of view than the base case standalone CO_2_ electrolyzer (Case C) where a syngas TCO of 1.4 €/kgSG had been estimated (Figure 4).

**Figure 6:**
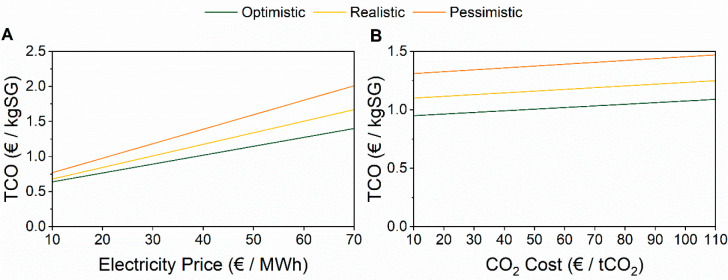
Sensitivity of syngas TCO [€/kg] to electricity (A) and CO_2_ (B) purchase price.

Finally, a study on the TCO sensitivity toward electricity and CO_2_ costs was carried out (Figure 6a,b, respectively). The gap between the different cases would be minimized for electricity prices below 10 €/MWh for which a syngas TCO of 0.7 €/kgSG can be achieved (Figure 6a). This price may become a reality for specific geographies and highly intermittent power supply. It seems, however, unrealistic in the short to medium term if high renewable power availability is required. As opposed to the electricity price, the CO_2_ purchase cost is shown to have a significantly lower impact on the final TCO of the syngas, which is an important observation for the future adoption of DAC technologies (Figure 6b). Even under optimistic conditions, the syngas produced by means of CO_2_ electrolysis is still less competitive from an economic standpoint than traditional methods. Syngas produced through coal gasification or methane reforming leads to prices between 0.5 and 0.7 €/kgSG.^71^ This difference calls for legislation and initiatives to promote the use of renewable syngas and bridge the gap of the green premium.

## Summary and Outlook

Since many industries rely on a consistent supply of syngas, the electrochemical reduction of CO_2_ and water to syngas can contribute to their transformation to become more sustainable and eventually carbon negative.^72^ Electrochemical approaches can also alter the dynamics of the syngas market, as smaller, decentralized solutions can emerge to be deployed at a customer’s facility, converting CO_2_ emission into value, saving on transportation cost, as well as reducing emission.^29^ By transforming syngas into synthetic fuels by Fischer–Tropsch or other catalytic processes, it can be integrated into existing infrastructure, significantly reducing investment costs compared to other approaches of power-to-gas solutions.^73^

In this Perspective, we have shown that syngas production from a stand-alone CO_2_ electrolyzer system would be possible from a technical point of view. However, it appears that it would not be reasonable from an energetic and therefore economical point of view. Through the combination of experimental data and techno-economic analysis, we have shown that the production of a syngas is always significantly less expensive when a CO_2_ electrolyzer is operated with the final goal of having only CO as the final product and then coupled with a PEM electrolyzer for H_2_ supply. We conclude that future studies shall focus on achieving high CO Faradaic efficiencies (over 98%). This selectivity shall be achieved at industrially relevant current densities (>400 mA/cm^2^), and degradation rates must be further minimized. To reach these key performance indicators, research and development on MEAs and electrolyzer cell/stacks shall go hand-in-hand because they mutually affect their applicability. Our study also indicated that downstream separation of O_2_ and CO from residual CO_2_ significantly contributes to the final investment costs (about 7–14%). Therefore, development, optimization, and/or integration of the gas treatment at the anode and cathode sides will also play an important role in further decreasing the total TCO of CO. Gaining substantial operational experience at a relevant scale will be key to allow the commercialization of CO_2_ electrolysis to CO. Large demonstration projects supported by funding agencies would therefore be the next natural step in the commercial development of CO_2_ electrolysis for CO and syngas when coupled with water electrolysis.

## Supporting Information Available

Literature review of a CO_2_ electrolyzer stack costs, selected examples from CO_2_ electrolysis studies, TEA methodology, additional parameters for market analysis, details of the purification model, and energy consumption data in the different scenarios (PDF)
