# Supplementary material for: Renewable Syngas Generation via Low-Temperature Electrolysis: Opportunities and Challenges
Source: ACS Energy Lett. 2023 Dec 29;9(1):288–97. doi: 10.1021/acsenergylett.3c02446 (PMC10795495; doi:10.1021/acsenergylett.3c02446)
Supplement: Supplementary file 1 — nz3c02446_si_001.pdf [file nz3c02446_si_001.pdf]

Supporting Information

Renewable Syngas Generation via Low-Temperature Electrolysis: Opportunities and Challenges

Andrés Raya-Imbernón[1], Angelika A. Samu[2,3], Stefan Barwe[1], Giuseppe Cusati[1], Tamás Fődi[2], Balázs M. Hepp[2], and Csaba Janáky[2,3]*

[1]Air Liquide Forschung & Entwicklung GmbH, Innovation Campus Frankfurt, Gwinnerstraße 27–33, 60388 Frankfurt am Main, Germany.,

[2]eChemicles Zrt, Alsó Kikötő sor 11, Szeged, H-6726, Hungary

[3]Department of Physical Chemistry and Materials Science, University of Szeged, Rerrich Square 1, Szeged, H-6720, Hungary

*Corresponding authors: giuseppe.cusati@airliquide.com, janaky@chem.u-szeged.hu

S1: Electrochemical Performance of CO_2 electrolyzer

Table S1. Selected examples from CO_2 electrolysis studies for producing CO and syngas.

Cell type	Cathode catalyst	Anode catalyst	Current density	Electrode potential / cell voltage	H_2/CO ratio	Ref.
H-cell	Au + Cu (1/3, 2/3, 3/3 monolayer)	Pt mesh	- Au + 1/3 Cu monolayer → 23 mA cm^{-2}<br>- Au + 2/3 Cu monolayer → 21 mA cm^{-2}<br>- Au + 3/3 Cu monolayer → 19 mA cm^{-2}	-0.65 V vs. RHE	- Au + 1/3 Cu monolayer → 0.5<br>- Au + 2/3 Cu monolayer → 1<br>- Au + 3/3 Cu monolayer → 1.5	1
	Lattice prefection ZnO<br>Lattice dislocation ZnO	Pt foil	- Lattice prefection ZnO → 21 mA cm^{-2}<br>- Lattice dislocation → 23 mA cm^{-2}	-1.4 V vs. RHE	- Lattice prefection ZnO → 2.83<br>- Lattice dislocation ZnO → 0.47	2
	- F-γ-In_2Se_3<br>- P-γ-In_2Se_3	Pt gauze	- F-γ-In_2Se_3 → 62 mA cm^{-2}<br>- P-γ-In_2Se_3 → 44 mA cm^{-2}	-2.0 V vs. SCE	- F-γ-In_2Se_3 → 0.04<br>- P-γ-In_2Se_3 → 0.82	3
	N-doped tubular carbon foam	Pt foil	- CF-30 → 9 mA cm^{-2}<br>- CF-60 → 11 mA cm^{-2}<br>- CF-90 → 10 mA cm^{-2}<br>- CF-120 → 16 mA cm^{-2}	-0.6 V vs. RHE	- CF-30 → 2.86<br>- CF-60 → 1.89<br>- CF-90 → 0.95<br>- CF-120 → 0.53	4
	40 wt% Pd/C	Pt mesh	4.4 mA cm^{-2}	-1.0 V vs. RHE	4.35	5
	Zn	n/a	- Zn-1 → 13 mA cm^{-2}<br>- Zn-2 → 11 mA cm^{-2}<br>- Zn-3 → 12 mA cm^{-2}	-0.9 V vs. RHE	- Zn-1 → 2.22<br>- Zn-2 → 1.27<br>- Zn-3 → 0.88	6
Flow cell	Ag	Ni mesh	150 mA cm^{-2}	-1.2 V vs. RHE	0.11	7
	Ag	IrO_2	342 mA cm^{-2}	-2.75 V	0.05	8
MEA-type	Ag	IrO_2	100 mA cm^{-2}	-3.0 V	0.01	9
	Au<br>AgAu	IrO_2	200 mA cm^{-2}	- 400 nm Au → 3.54 V<br>- 400 nm npAu → 3.22 V<br>- 800 nm npAu → 3.18 V	- 400 nm Au → 3.35<br>- 400 nm npAu → 1.86<br>- 800 nm npAu → 1.63	10
	Ag	IrO_2	150 mA cm^{-2}	-3.16 V	0.02	11
	Ag	IrO_2	600 mA cm^{-2}	-3.3 V	0.05	12
	Ag	IrO_2	300 mA cm^{-2}	-3.0 V	0.18	13
	Ag	IrO_2	- FRG H23C6 → 580 mA cm^{-2}<br>- FRG H23C2 → 520 mA cm^{-2}<br>- FRG H23I2 → 540 mA cm^{-2}<br>- SGC 39AA → 320 mA cm^{-2}	-3.0 V	- FRG H23C6 → 0.05<br>- FRG H23C2 → 0.4<br>- FRG H23I2 → 0.8<br>- SGC 39AA → 4.0	14

S2. Observed, future predicted, and theoretical single pass conversions for the different scenarios.

The theoretical single-pass CO_2 conversion naturally decreases by increased HER. This is because OH^- ions form during both CO_2 and H_2O reduction at the cathode, which results in carbonate formation in a CO_2 rich environment. This leads to a more pronounced CO_2 crossover in a form of carbonate conduction (Table S2).

Table S2. Summary of single-pass CO_2 conversion values.

Scenario	Theoretical Max. single-pass CO_2conversion [%]	Single-pass CO_2 conversion (Today) [%]	Single-pass CO_2 conversion (2030) [%]
A (pessimistic)	50	25	30
A (realistic)	50	40	40
A (optimistic)	50	40	45
B	33	25	28
C	25	15	18

S3 CO_2 Electrolyser Stack and System

The TCO is the overall cost of a product throughout its life cycle and includes all fixed and variable costs of a given process. The capital expenditures (CAPEX) encompass the capital investment and its depreciation, the maintenance required, the insurance costs, the stack replacement and a given profit margin. The operating expenditures (OPEX) involve feedstocks and utilities necessary to manufacture the targeted product. In our case, the variable costs are mainly the electricity and the CO_2 purchase needed. Knowing the TCO by performing a careful techno-economic assessment (TEA) provides additional opportunities for value creation and several additional benefits such as identifying cost drivers, recognizing areas of improvement, and pinpointing competitive advantages.

A wide range of values for the anion exchange membrane (AEM) stack costs depending on the assumptions made can be found in the literature for CO_2 electrolysis. The main challenge when using costs coming from literature review is the incomplete information leading to more inaccuracy. Table S3 summarizes the available cost information found screening different sources. The water electrolysis industry has become accustomed to speaking of costs in €/kW. This metric is used to compare different types of electrolyzers. This is possible because most water electrolyzers operate at voltages between 1.8 and 2 V.[15,16] However, CO_2 electrolysis operates at different conditions, so the use of the same metric can lead to confusion. In order to reliably compare different data sources, the conversion from €/kW to €/m^2 has been performed (Eq. 1). All values have been adjusted for inflation using the CEPCI index methodology.[17]

$$\text{Stack Cost } [€/m^2] = \text{Stack Cost } [€/kW] \times V\,[V] \times J\,[A/m^2] / 1000 \quad \text{Eq. 1}$$

As it can immediately be seen in Table S2, the stack cost significantly depends on the designed operational parameters and a wide range of values can be found in literature. For this reason, in this paper, a so-called bottom-up approach has been also employed for calculating the stack cost of an AEM CO_2 electrolyzer. According to the benchmarking performed based on the similarities between AEM and PEM electrolyzers, our bottom-up approach encompasses most costs providing an estimation for the prevalent components of an AEM stack. Although AEM is currently considered immature with low TRL compared to similar technologies, it is expected that it will mature in a growing pace.[18,19] Compared to the similar PEM technology, costs are

expected to be lower due to several factors: no platinum is needed, while less Iridium is required based on innovative immobilization methods, as well as novel production methods such as metal stamping and sintered meshes are expected to drive the costs further down.[20,21]

This approach (Table S4) gives a non-installed stack cost which is within the range of the values found in the literature. However, it must be kept in mind that this final figure should be seen as a target cost in 2030 for a 10 MW stack (at a reference voltage and current of 2.6 V and 500 mA/cm^2). An installation factor of 1.6 is used for the electrolyzer system.[16]

Understanding the effect of equipment size on cost is of paramount importance as usually large chemical plants profit from the economy of scale. In the chemical industry scaling factors around 0.7 are often applied to the main equipments.[22] However, electrolyzers cannot be estimated under the same rules. There are limitations on the sizes that stacks can reach. Beyond a specific size, any increase in capacity needs the use of multiple stacks in parallel, so that for large installed capacities it is a simple numbering up of the same stack.

The balance of plant (BoP) on the other hand, does have scaling factors that can be applied to it. BoP is divided into power electronics, gas conditioning and electrolyte management, and each of these components would have its own factor.[18,23] However to simplify the calculation it is assumed that for large capacities there is no economy of scale. The scaling factor applied to the BoP is therefore equal to 1.

Table S3. Literature Review of a CO_2 Electrolyser Stack Cost.

**Stack Cost (RY)**	**Voltage**	**Current**	**Stack Cost (2023)**		**Reference**
**$/kW**	**(V)**	**(mA/cm^2)**	**€/kW**	**€/m^2**	
175	1.8	800	216	3107	[20]
174	1.8	1500	215	5792	[20]
550	1.75	400	570	3993	[24]
400	2.3	200	518	2383	[25]
153	2	300	199	1192	[25]
87	1.84	1000	78	1442	[26]
110	1.74	1500	99	2586	[26]

* RY: Reference Year

* €/$ (03 August 2023): 0.91

Table S4. Bottom-up Approach for the Estimation of the CO_2 Electrolyser Stack Price.

Stack cost estimation (€/m²)	eChemicles estimate			
	2030		2040	
**Capacity**	**10 MW**	**200 MW**	**10 MW**	**200 MW**
AEM + catalysts	1000	813	613	442
Anode PTL	563	513	450	425
Cathode PTL	150	125	113	113
BPP	263	225	200	175
BPP Coating	391	191	319	156
Frame + sealing	111	36	149	61
Pressure plate	59	50	58	50
BoP Stack	20	12	21	17
Assembly	33	13	21	11
**SUM**	**2587**	**1976**	**1944**	**1450**

S4: Purification Scheme

Due to the incomplete CO_2 per pass conversion at the cathode and the CO_2 evolution at the anode because of the carbonate migration, CO_2 must be separated from CO (and eventually H_2) and O_2 and recycled into the electrolyzer to minimize variable costs and carbon footprint.

There are many ways described in the literature to separate CO_2 from O_2, and CO_2 from CO and H_2. The most common one is the Pressure Swing Adsorption which is already widely used in the purification of H_2 and biomethane.

To determine the TIC of the PSAs a literature review has been carried out. Different sources have been found in the field of biogas upgrading and the costs found have been adapted to inflation using the CEPCI index methodology (Table S5).[17] For sake of simplification the same costs and efficiencies for the PSAs at the anode and at the cathode have been assumed.

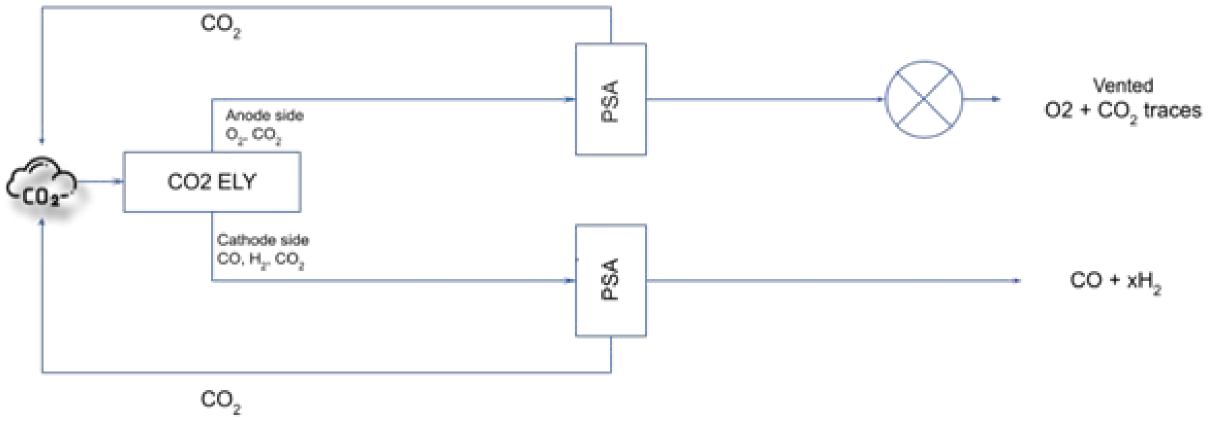

Figure S1: Block Flow Diagram of the Purification Scheme Considered of Syngas.

As the data collected corresponds to much lower capacities than those to be handled in this study (Table S6) and the amount of data available is not sufficient to establish a reliable regression (Figure S2), a price of 2.58M€ for a PSA with a capacity of 1000 Nm^3/h has been used and a scaling factor of 0.7 has been applied. This is also in line with other several TEA studies performed on CO_2 electrolysis.[24,25,27]

Table S5. Literature Review of a PSA Total Installed Cost.

Flow Rate (m^3/h)	TIC	Currency	Year	TIC (€ 2023)	**Reference**
1000	1990000	$	2017	2.58	[25]
1400	3156840	€	2020	4.47	[28]
2000	2400000	€	2013	3.38	[29,30]
1250	2187500	€	2013	3.08	[29,30]
500	1500000	€	2013	2.11	[29,30]

* €/$ (03 August 2023): 0.91

Table S6 Total Flow Rates to be treated by PSA system at the anode and cathode for each syngas production route.

	**A**	**B**	**C**
Flow Rate Cathode (Nm^3/h)	15,000	30,000	56,667
Flow Rate Anode (Nm^3/h)	15,306	30,000	44,999

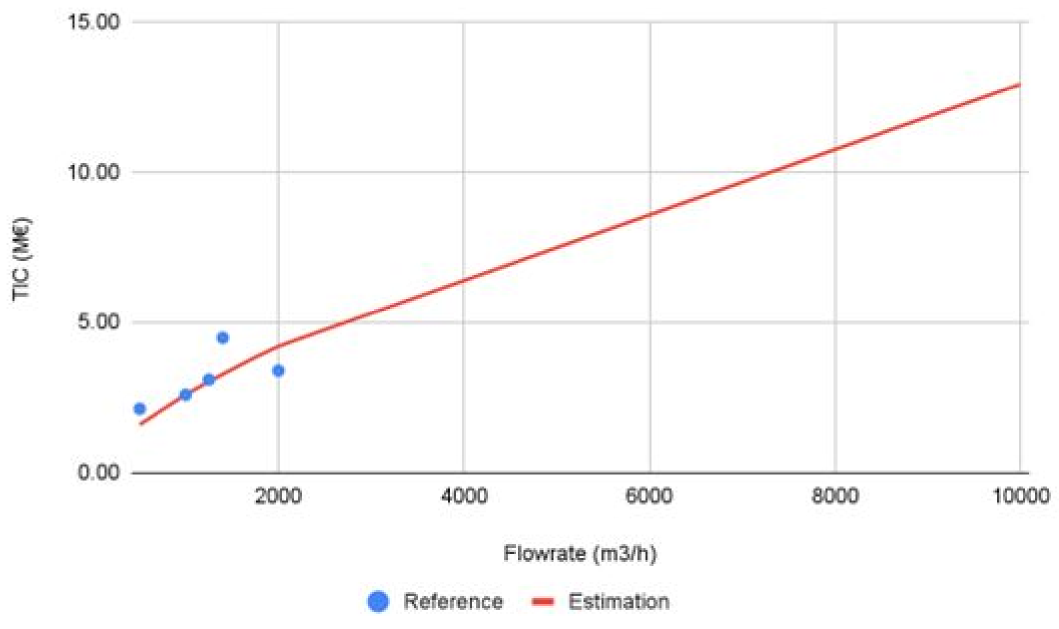

Figure S2: Correlation Between the Flow Rate [Nm^3/h] processed by a PSA System and its Corresponding TIC [M€]. Reference values vs Assumption.

S5: Carbon-monoxide demand estimation

Based on the different estimations available on syngas and CO-based products, an approximate demand for CO has been presented considering its main applications (excluding energy applications). Table S7 presents the product market size as well as the required syngas (or pure CO)* to supply given market demands regarding the chemical sector.

Table S7. *Syngas and CO uptake estimate based on their main applications.

**Product**	**Methanol**[31]	**Butanol**[32]	**DME**[33]	**Acetic acid**[34]	**MDI**[35]	**TDI**[36]	**Polycarbonates**[37]
**Market size (Mt)**	90	6	4.5	17.5	7	2.4	4.9
**Syngas* (Mt)**	90	2.6	6.26	9.3	1.6	0.8	0.12

The chemicals sector is projected to utilize more than 170 Mt of CO by 2030. Syngas witnesses a wide range of applications in the field of industrial chemicals, all of which are expected to increase during the forthcoming years, significantly contributing to the growth of its demand. For example, according to the Global CO_2 Initiative, methanol in itself is expected to quadruple from 2020 to 2050 in the volume of the total addressable market.[38,39]

Steel and iron production is also an industry looking towards the utilization of CO, especially in the form of direct reduced iron (DRI) production. According to estimates, DRI can take up more than 20 Mt of CO by 2050.[40]

Furthermore, as synthetic fuels gain traction it is also expected to be a significant consumer of CO due to tightening regulations facilitating the adoption of sustainable aviation fuels, with an increasing share of renewable fuels of non-biological origin (RFNBO). Assuming that CO based e-fuels will make up 10% of SAF production in 2030, it could result in a demand of more than 6 Mt CO only for SAF production via the Fischer-Tropsch process. In line with the projected growth of the jet market, SAF will need at least 300 Mt CO by 2050. [41–43]

S6: Energy consumption in the different scenarios

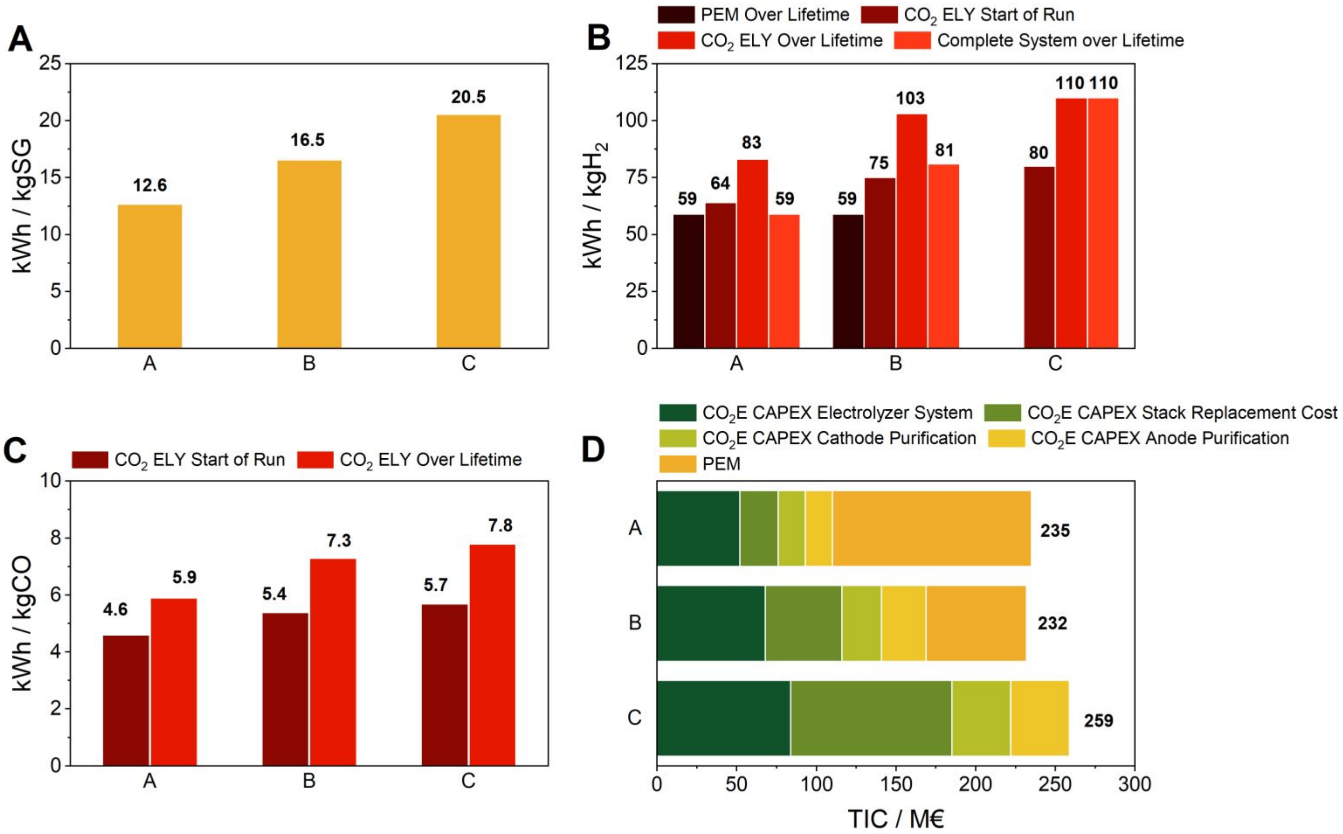

Figure S3. Energy Consumption [kWh/kgSG] of the Overall System (a), energy consumption allocation of the CO_2 electrolyzer and the overall system for H_2 (b) and CO (c). TIC [M€] of the three cases studies (d).

S7: Parameters for optimistic/realistic/pessimistic scenario analysis

Table S8. Electrochemical performance parameters for CO_2 electrolysis for case A Pessimistic, Base and Optimistic Scenarios for 2030. PEM water electrolysis performance parameters were kept constant.

	**Pessimistic**	**Base**	**Optimistic**
FE_{CO} (%)	95%	98%	99%
Current Density (mA/cm^2)	400	600	800
Voltage (V)	2.8	2.4	2.3
Single pass CO_2 conversion (%)	30%	40%	45%
Degradation Rate (μV/hours)	20	10	8

References

(1) Ross, M. B.; Dinh, C. T.; Li, Y.; Kim, D.; De Luna, P.; Sargent, E. H.; Yang, P. Tunable Cu Enrichment Enables Designer Syngas Electrosynthesis from CO_2. *J Am Chem Soc* **2017**, *139* (27), 9359–9363.

(2) Qin, B.; Zhang, Q.; Li, Y. H.; Yang, G.; Peng, F. Formation of Lattice-Dislocated Zinc Oxide via Anodic Corrosion for Electrocatalytic CO_2 Reduction to Syngas with a Potential-Dependent $CO:H_2$ Ratio. *ACS Appl Mater Interfaces* **2020**, *12* (27), 30466–30473.

(3) Yang, D.; Zhu, Q.; Sun, X.; Chen, C.; Guo, W.; Yang, G.; Han, B. Electrosynthesis of a Defective Indium Selenide with 3D Structure on a Substrate for Tunable CO_2 Electroreduction to Syngas. *Angewandte Chemie - International Edition* **2020**, *59* (6), 2354–2359.

(4) Li, H.; Xiao, N.; Wang, Y.; Li, C.; Ye, X.; Guo, Z.; Pan, X.; Liu, C.; Bai, J.; Xiao, J.; Zhang, X.; Zhao, S.; Qiu, J. Nitrogen-Doped Tubular Carbon Foam Electrodes for Efficient Electroreduction of CO_2 to Syngas with Potential-Independent CO/H_2 Ratios. *J Mater Chem A Mater* **2019**, *7* (32), 18852–18860.

(5) Sheng, W.; Kattel, S.; Yao, S.; Yan, B.; Liang, Z.; Hawxhurst, C. J.; Wu, Q.; Chen, J. G. Electrochemical Reduction of CO_2 to Synthesis Gas with Controlled CO/H_2 Ratios. *Energy Environ Sci* **2017**, *10* (5), 1180–1185.

(6) Qin, B.; Li, Y.; Fu, H.; Wang, H.; Chen, S.; Liu, Z.; Peng, F. Electrochemical Reduction of CO_2 into Tunable Syngas Production by Regulating the Crystal Facets of Earth-Abundant Zn Catalyst. *ACS Appl Mater Interfaces* **2018**, *10* (24), 20530–20539.

(7) Dinh, C. T.; García De Arquer, F. P.; Sinton, D.; Sargent, E. H. High Rate, Selective, and Stable Electroreduction of CO_2 to CO in Basic and Neutral Media. *ACS Energy Lett* **2018**, *3* (11), 2835–2840.

(8) Verma, S.; Lu, X.; Ma, S.; Masel, R. I.; Kenis, P. J. A. The Effect of Electrolyte Composition on the Electroreduction of CO_2 to CO on Ag Based Gas Diffusion Electrodes. *Physical Chemistry Chemical Physics* **2016**, *18* (10), 7075–7084.

(9) Lee, W. H.; Ko, Y. J.; Choi, Y.; Lee, S. Y.; Choi, C. H.; Hwang, Y. J.; Min, B. K.; Strasser, P.; Oh, H. S. Highly Selective and Scalable CO_2 to CO - Electrolysis Using Coral-Nanostructured Ag Catalysts in Zero-Gap Configuration. *Nano Energy* **2020**, *76*, 105030.

(10) Qi, Z.; Kashi, A. R.; Buckley, A. K.; Miller, J. S.; Ye, J.; Biener, M. M.; Foucher, A. C.; Stach, E. A.; Ma, S.; Kuhl, K. P.; Biener, J. Effect of Gold Catalyst Surface Morphology on Wetting

Behavior and Electrochemical CO_2 Reduction Performance in a Large-Area Zero-Gap Gas Diffusion Electrolyzer. *Journal of Physical Chemistry C* **2022**, *126* (46), 19637–19646.

(11) Larrazábal, G. O.; Strøm-Hansen, P.; Heli, J. P.; Zeiter, K.; Therkildsen, K. T.; Chorkendorff, I.; Seger, B. Analysis of Mass Flows and Membrane Cross-over in CO_2 Reduction at High Current Densities in an MEA-Type Electrolyzer. *ACS Appl Mater Interfaces* **2019**, *11* (44), 41281–41288.

(12) Liu, Z.; Yang, H.; Kutz, R.; Masel, R. I. CO_2 Electrolysis to CO and O_2 at High Selectivity, Stability and Efficiency Using Sustainion Membranes. *J Electrochem Soc* **2018**, *165* (15), J3371–J3377.

(13) Endrödi, B.; Kecsenovity, E.; Samu, A.; Darvas, F.; Jones, R. V.; Török, V.; Danyi, A.; Janáky, C. Multilayer Electrolyzer Stack Converts Carbon Dioxide to Gas Products at High Pressure with High Efficiency. *ACS Energy Lett* **2019**, *4* (7), 1770–1777.

(14) Samu, A. A.; Szenti, I.; Kukovecz, Á.; Endrődi, B.; Janáky, C. Systematic Screening of Gas Diffusion Layers for High Performance CO_2 Electrolysis. *Commun Chem* **2023**, *6* (1), 41.

(15) Taibi, E.; Blanco, H.; Miranda, R.; Carmo, M. Green Hydrogen Cost Reduction Scaling Up Electrolysers to Meet The 1.5°C Climate Goal; International Renewable Energy Agency **2020**.

(16) Van't Noordende, H.; Ripson, P. Baseline Design and Total Installed Costs of a GW Green Hydrogen Plant. State-of-the-Art Design and Total Installed Capital Costs; Report **2020**, https://ispt.eu/publications/public-report-gigawatt-green-hydrogen-plant/ (accessed 2023-08-18).

(17) Mignard, D. Correlating the Chemical Engineering Plant Cost Index with Macro-Economic Indicators. *Chemical Engineering Research and Design* **2014**, *92* (2), 285-294.

(18) Holst, M.; Aschbrenner, S.; Smolinka, T.; Voglstätter, C.; Grimm, G. Cost Forecast for Low-Temperature Electrolysis-Technology Driven Bottom-Up Prognosis for PEM and Alkaline Water Electrolysis Systems; Report **2021,** https://publica.fraunhofer.de/entities/publication/5a0b888c-453c-449b-9dbb-d18f61c3abee/details (accessed 2023-12-08).

(19) Küngas, R. Review - Electrochemical CO_2 Reduction for CO Production: Comparison of Low- and High-Temperature Electrolysis Technologies. *J Electrochem Soc* **2020**, *167* (4), 044508.

(20) Toussi, O. Hydrogen Production Cost by AEM Water Electrolysis **2020**, www.ionomr.com.

(21) Mayyas, A.; Ruth, M.; Pivovar, B.; Bender, G.; Wipke, K. Manufacturing Cost Analysis for Proton Exchange Membrane Water Electrolyzers **2019,** https://www.nrel.gov/docs/fy10osti/72740.pdf.

(22) Carberry, J. J.; Walker, W. H.; White, A. H.; Jackson, D. D.; James, J. H.; Lewis, W. K.; Curtis H C Parmelee, H. A. Plant Design and Economics for Chemical Engineers.

(23) Böhm, H.; Zauner, A.; Rosenfeld, D. C.; Tichler, R. Projecting Cost Development for Future Large-Scale Power-to-Gas Implementations by Scaling Effects. *Appl Energy* **2020**, 264, 114780.

(24) Shin, H.; Hansen, K. U.; Jiao, F. Techno-Economic Assessment of Low-Temperature Carbon Dioxide Electrolysis. *Nat Sustain* **2021**, *4* (10), 911–919.

(25) Jouny, M.; Luc, W.; Jiao, F. General Techno-Economic Analysis of CO_2 Electrolysis Systems. *Ind Eng Chem Res* **2018**, *57* (6), 2165–2177.

(26) James, D. B.; Huya-Kouadio, J. M.; Houchins, C.; Acevedo, Y.; McNamara, K.; Saur, G. Hydrogen Production Cost and Performance Analysis.

(27) Thomas, M.; Diego, I. O.; Wenqin, L.; et al. Electrolyzer Energy Dominates Separation Costs in State-of-the-Art CO_2 Electrolyzers: Implications for Single-Pass CO_2 Utilization. *Joule* **2023**, *7* (4), 782-796.

(28) Kohlheb, N.; Wluka, M.; Bezama, A.; Thrän, D.; Aurich, A.; Müller, R. A. Environmental-Economic Assessment of the Pressure Swing Adsorption Biogas Upgrading Technology. *Bioenergy Res* **2021**, *14* (3), 901–909.

(29) Sánchez-Martín, L.; Ortega Romero, M.; Llamas, B.; Suárez Rodríguez, M. del C.; Mora, P. Cost Model for Biogas and Biomethane Production in Anaerobic Digestion and Upgrading. Case Study: Castile and Leon. *Materials* **2023**, *16* (1), 359.

(30) Fredric, B.; Christian, H.; Tobias, P.; Daniel, T. Biogas Upgrading - Review of Commercial Technologies; Report **2012**, https://portal.research.lu.se/en/publications/biogas-upgrading-review-of-commercial-technologies (accessed 2023-08-18).

(31) Methanol Market Analysis: Industry Market Size, Plant Capacity, Production, Operating Efficiency, Demand & Supply, End-User Industries, Sales Channel, Regional Demand, Company Share, Foreign Trade, 2015-2032 **2023**, https://www.chemanalyst.com/industry-report/methanol-market-219 (accessed 2023-08-18).

(32) AgileIntel Research. Market Volume of N-Butanol Worldwide from 2015 to 2022, with a Forecast for 2023 to 2030; Report **2023**, https://www.statista.com/statistics/1245211/n-butanol-market-volume-worldwide/ (accessed 2023-08-18).

(33) Dimethyl Ether Market Size & Share Analysis - Growth Trends & Forecasts (2023 - 2028); Report ID: SR112023A2224, **2023** https://www.imarcgroup.com/dimethyl-ether-market (accessed 2023-08-18).

(34) AgileIntel Research. Market Volume of Acetic Acid Worldwide from 2015 to 2022, with a Forecast for 2023 to 2030; Report, **2023** https://www.statista.com/statistics/1245203/acetic-acid-market-volume-worldwide/ (accessed 2023-08-18).

(35) Methylene Diphenyl Di-Isocyanate (MDI) Market - Growth, Trends, COVID-19 Impact, and Forecasts (2023-2028); Report ID: 4520097 **2023**, https://www.researchandmarkets.com/reports/4520097/methylene-diphenyl-di-isocyanate-mdi-market.

(36) Toluene Diisocyanate Market Size & Share Analysis - Growth Trends & Forecasts (2023 - 2028); Report ID:6240330, **2023**, https://www.mordorintelligence.com/industry-reports/toluene-diisocyanate-market (accessed 2023-08-18).

(37) Global Polycarbonate Market Size, Share, Trends, Growth, Forecast, Price, Outlook: By Application: Electrical and Electronics, Automotive, Construction, Optical Media, Packaging, Consumer, Medical; Regional Analysis; Historical Market and Forecast (2018-2028); SWOT Analysis; Competitive Landscape; Industry Events and Developments; Report ID: GVR-1-68038-269-3 **2022**, https://www.expertmarketresearch.com/reports/polycarbonate-market (accessed 2023-08-18).

(38) Syngas Market: Global Industry Trends, Share, Size, Growth, Opportunity and Forecast 2023-2028; Report ID: SR112023A1555, **2022** https://www.imarcgroup.com/syngas-market (accessed 2023-12-07).

(39) Sick, V.; Stokes, G.; Mason, F.; Yu, Y.-S.; Van Berkel, A.; Daliah, R.; Gamez, O.; Gee, C.; Kaushik, M. Implementing CO_2 Capture and Utilization at Scale and Speed; Report **2022**, https://deepblue.lib.umich.edu/handle/2027.42/174094 (accessed 2023-12-08).

(40) Chris Barrington. The Iron Ore Challenge for Direct Reduction On Road to Carbon-Neutral Steelmaking. https://www.midrex.com/tech-article/the-iron-ore-challenge-for-direct-reduction-on-road-to-carbon-neutral-steelmaking/ (accessed 2023-12-07).

(41) International Civil Aviation Organization. Short-Term Projections on SAF Production; Report **2023**, https://www.icao.int/environmental-protection/Documents/SAF/ICAO%20SAF%20short-term%20projections%20-%20methodology%20and%20results.pdf (accessed 2023-08-18).

(42) International Civil Aviation Organization. Enviromental Trends in Aviation to 2050; Report **2023**, https://www.icao.int/environmental-protection/Documents/EnvironmentalReports/2019/ENVReport2019_pg17-23.pdf (accessed 2023-08-18).

(43) European Regions Airline Association. Council and Parliament Reach Agreement- on Refueleu Aviation. https://www.eraa.org/council-and-parliament-reach-agreement-refueleu-aviation (accessed 2023-09-27).
